# Worldwide news and comment

**DOI:** 10.1136/tc-2023-058403

**Published:** 2023-10-19

**Authors:** Karen Evans-Reeves, Ruth Canty, Manuja Niranshi Perera

**Affiliations:** 1 Department for Health, University of Bath, Bath, UK; 2 Global and Tropical Health, Menzies School of Health Research, Casuarina, Northern Territory, Australia; 3 Department of Public Health, University of Kelaniya Sri Lanka, Ragama, Western, Sri Lanka; 4 Centre for Combating Tobacco Sri Lanka, Colombo 05, Sri Lanka

**Keywords:** Addiction, Advertising and Promotion, Advocacy

## Asia

### Bangladesh: tobacco tax system needs urgent reform

Bangladesh is globally the ninth largest cigarette market. Adolescent (13–15 year olds) smoking prevalence is 9.2%, and over 35% of those aged over 15 are currently smoking. Tobacco use claims 161 000 lives a year and incapacitates hundreds of thousands of people. A 2019 study conducted by the American Cancer Society put the financial burden of tobacco use in Financial Year (FY) 2017–18 at 305.6 billion Bangladeshi Taka (BDT) much higher than the revenue generated from tobacco which was BDT 228.1 billion. Overall, tobacco use has emerged as a growing threat to the public health, economy, and environment of the country. For this reason, the country has pledged to become a tobacco-free country by 2040.

Yet Bangladesh’s convoluted and flawed tobacco tax structure is greatly at odds with this vision. Its tax system is ineffective in discouraging and reducing tobacco use and therefore incongruous with Article 6 of the WHO Framework Convention on Tobacco Control (FCTC). Article 6 requires parties to adopt the simplest and most effective tobacco tax system which will contribute to reducing tobacco use, an obligation Bangladesh is yet to fulfil, despite the Prime Minister’s 2016 directive to simplify and strengthen the existing tax system.

A close look at the existing tobacco tax structure of Bangladesh reveals several debilitating features that provide no benefit to the country, its government, or its people—only to the tobacco industry.

### Weak and flawed tax base fails to reduce use and raise revenue

Bangladesh considers the retail prices of tobacco products as its tax base and a supplementary duty (excise tax) is imposed as a percentage of the price. Since the retail prices of tobacco in Bangladesh remain low, the tax does little to control tobacco use or raise revenue. An analysis of WHO data for cigarette prices in 176 countries shows that a pack of 20 sticks of the most sold cigarette brand costs (International dollar) the most in Sri Lanka, US$ 24.92 and the tax rate imposed is 72.02%. However, despite Bangladesh’s tax rate at 73% being higher than Sri Lanka, a pack of 20 cigarettes is sold for just US$ 2.91.

This paradox of having low prices despite high taxation is, in fact, the result of a flawed tax system that is being exploited by tobacco companies to maximise their profits, while denying government the benefits effective tobacco taxes can provide. The budgetary decision of not raising taxes in the low tier in recent fiscal years has allowed tobacco companies to maximise their profits. Raising the base prices alone (with no change in the percentage of supplementary duty) allows companies to pocket a significant share of the increased price.

### A convoluted and flawed tobacco tax structure

Bangladesh currently has a multi-tiered (low, medium, high, and premium) ad valorem tax structure for cigarettes. The National Board of Revenue reported that in FY 2006–07, only 25% of cigarette smokers smoked low-tier cigarettes, but this percentage jumped to 71 % in FY 2017 – 18. This is a result of consistently low tax in this tier which allowed companies to introduce new low-tier cigarette brands. Thus, those who may have quit due to higher prices responded by switching to low-tier brands.

### A tobacco tax regime with narrow coverage and weak administration

Jarda, gul, bidi and other cheap tobacco products have largely remained out of the tax net and are produced locally without almost any government supervision. While more than 50% of the country’s total tobacco users use are also users of smokeless tobacco (SLT) products, the revenue contribution of these products remains at only 0.15% of total tobacco revenue in FY 2020–21. As the majority of SLT users are women and those living in poverty, tobacco taxation has largely failed to safeguard the most vulnerable populace.

The result of such convoluted tax structure is already evident. According to a 2021 WHO report, Bangladesh ranks 107th among 167 countries where cigarettes are sold cheaply (with 167th being the cheapest). In Southeast Asia, Bangladesh sells the second cheapest cigarette brands, after Myanmar.

### Increasing affordability

PROGGA (Knowledge for Progress), a Bangladesh-based research and advocacy organisation, analysed affordability in different tiers of cigarettes using the Relative Income Price (RIP) method. In FY 2015–16, a person who smokes cigarettes had to spend 7.78, 5.39, and 3.47% of their per capita share of gross domestic product (GDP) to buy 1000 sticks of premium, high, and medium tier cigarette brands respectively. By FY 2021–22, the numbers became considerably lower, respectively 5.82, 4.40, and 2.72% (ie, consumers could purchase the same quantity of cigarettes while spending less than before). What is more concerning is the trend of increasing affordability in the lowest price tier of cigarettes as 71% of cigarette users (as of FY 2017–18) belong to this tier. In FY 2018–19, a purchaser of low-tier cigarette brands had to spend 1.96% of per capita GDP for 1000 sticks, declining to 1.68% by FY 2021–22. As a result of this decline in real prices, smoking prevalence has plateaued in recent years.

### Becoming cheaper than essential commodities

In addition to this growing affordability, cigarettes are becoming cheaper compared with essential commodities like flour, the price of which jumped by 71.7% between March 2022 and March 2023. Cigarettes have seen only a 2.56% hike, and the prices of other tobacco products such as jarda, gul and bidi have seen no changes for FY 2022–23.

### Way forward

Increasing prices of tobacco products through more effective taxation is likely to be the most cost-effective measure for a country like Bangladesh as it discourages consumption while generating revenue for the government. Adoption of a specific excise tax system like 65 other countries have including the Philippines, Nepal, Sri Lanka, and Malaysia, is advised. Another 63 countries, including neighbouring India and Thailand, have already opted for a hybrid tax regime which means the application of both specific and ad valorem tax systems. Tobacco control organisations in Bangladesh have long been urging the government to adopt a specific tax system for tobacco. In addition, the number of price tiers in cigarettes should be reduced from the existing four to one. Prices of tobacco products must be increased in line with inflation and increases in per capita income. The tax net of the government must be expanded, capturing particularly jarda, gul, bidi, and other unregistered factories. There is no alternative to strengthening tax administration if Bangladesh is to meet its tobacco-free goal.

Mahir Dyan Amin, independent public health policy researcher, Dhaka, Bangladesh.

ABM Zubair, Executive Director of PROGGA (Knowledge for Progress), Director of the Centre for Research and Advocacy to Fight Tobacco (CRAFT), Bangladesh progga.bd@gmail.com

Md. Shahedul Alam, Head of Research and Advocacy, PROGGA, Bangladesh

## India: mandatory anti-tobacco warnings on OTT platforms

India has taken a ground-breaking step in public health by becoming the first country to mandate anti-tobacco warnings and disclaimers on over-the-top (OTT) platforms such as Netflix and Amazon Prime. OTT platforms are digital services delivering multimedia content over the internet, bypassing traditional broadcasting avenues. The ruling means that the publishers of online content which displays tobacco products or use will have to display 30-second anti-tobacco messages at the beginning and in the middle of the content. This pioneering move in India aligns with the country’s commitment to curbing tobacco’s influence on digital platforms and protecting its population’s health.

Concerns over normalising and glamorising smoking, especially among youth, have grown due to tobacco imagery on media platforms, including OTT services. This normalisation contributes to preventable diseases and premature deaths linked to tobacco use in India with studies showing that media exposure to tobacco imagery affects attitudes and behaviours.

Vigilant monitoring and enforcement mechanisms are essential. Regular audits should confirm compliance with regulations. Non-compliance must result in appropriate penalties to deter violations and ensure accountability.

Supporting research initiatives to evaluate the impact of anti-tobacco warnings on OTT platforms is vital. Such research offers insights into the warnings' effectiveness in altering smoking-related attitudes and behaviours, allowing for necessary adjustments.

India’s innovative decision to enforce anti-tobacco warnings and disclaimers on OTT platforms represents a significant step toward safeguarding public health and counteracting tobacco imagery’s influence in the digital domain. By prioritising citizens' well-being over commercial interests, the government demonstrates its commitment to establishing a tobacco-free society and raising awareness about tobacco’s harms.

Dr. Srishti Nawani, Masters in Public Health student,

Department of Community Medicine & School of Public Health,

Post Graduate Institute of Medical Education and Research, Chandigarh, India srinawani.2011@gmail.com

Dr. (Prof.) Sonu Goel, Professor of Health Management,

Department of Community Medicine & School of Public Health,

Post Graduate Institute of Medical Education and Research, Chandigarh, India

### India: is bat losing its foothold stake in ITC?

**Figure 1:**
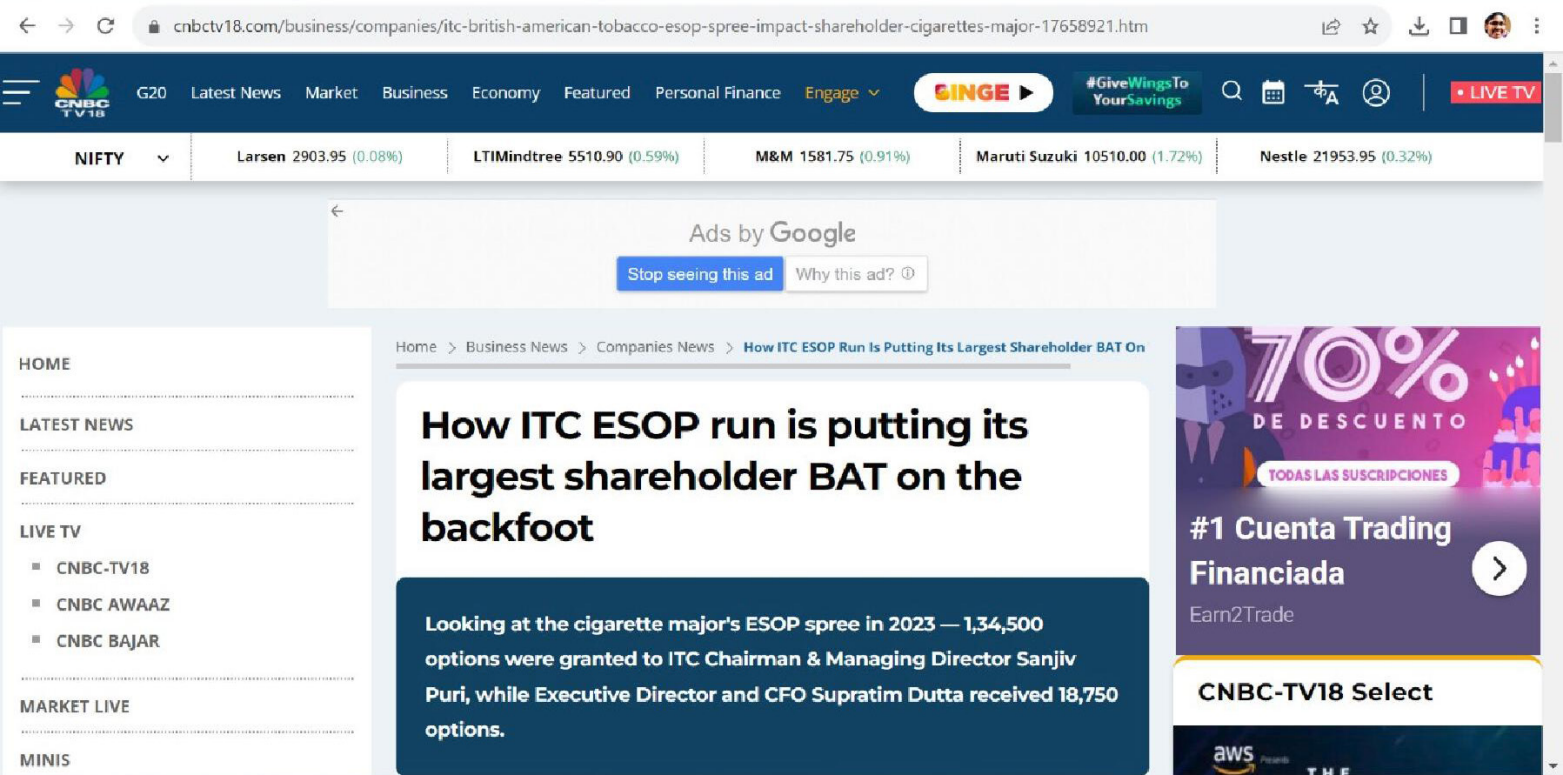
CNBC News story in India reporting that the ESOP move is detrimental to BAT.

Recent news coverage suggests that British American Tobacco (BAT) is losing its foothold in ITC (formally the India Tobacco Company), because of ITC's Employees’ Stock Option scheme (ESOP)(figure 1). The August 2023 announcement reported that BAT’s overall share of ITC is now less than 30%. Since the inception of the ESOP, BAT’s stake has reduced by just over 3% in absolute terms. It has been suggested that if BAT’s share drops below 25%, it will seriously compromise the company’s decision-making power within ITC. BAT has opposed the ESOP since 2018.

Established as the Imperial Tobacco Company of India Limited in 1910 by the British, ITC it was renamed the India Tobacco Company Limited in 1970. Since this time the company has been diversifying its business and to move away from its ‘tobacco’ related name tag the India Tobacco Company became ITC Limited in 1974. In 1975, ITC Limited entered the hospitality sector, its first actual diversification from tobacco related industries. This strategy was strongly opposed by BAT.

The Indian government, which also owns close to a third of ITC, was in favour of the diversification and subsequently banned foreign direct investments and pushed for the ESOP. BAT fought against it, suggesting that the government was acting inappropriately and denying the rights of corporations and consumers. ITC and BAT have been battling against each other for control for decades now. It remains to be seen what BAT’s next move will be.

## Europe

### Russia: PMI and JTI added to Ukrainian list of international sponsors of war

Following Russia’s invasion of Ukraine in February 2022, tobacco companies followed the lead of other Western companies in condemning Russia and declaring solidarity with the people of Ukraine. However, platitudes expressing concern about the suffering of the Ukrainian people from the tobacco industry ring somewhat hollow, given that in 2020 alone, more than 15 000 lung cancer deaths were reported in Ukraine.

We have previously reported here that in the wake of Russia’s invasion of Ukraine, while British American Tobacco and Imperial Tobacco had announced their intention to suspend sales of their products in Russia, Phillip Morris International and Japan Tobacco International (JTI) would continue, but had plans to scale back their involvement in the country. However, product sales for both companies have continued in Russia. The Kyiv School of Economics reported that JTI increased revenue in Russia in 2022 by USD$1.5 billion compared with 2021, with a total revenue of USD$7.4 billion, and paying USD$193 million in profit tax to the Russian Government. PMI’s revenues were USD$7.9 billion in 2022, resulting in USD$206 million in profit tax.

In August 2023, Ukraine’ s National Corruption Prevention Agency added PMI and JTI to its list of international sponsors of war, a list of companies who continue to do business in Russia and support the Russian economy. Russia is a lucrative market for the tobacco industry: it has a high smoking prevalence, is the fourth largest market in the world, and provides ample lobbying opportunities for the industry. Reuters reports that PMI and JTI have long-standing ties with Russia, with both companies purchasing 20% stakes in Megapolis, the tobacco distribution company owned by Russian billionaire Igor Kesaev in 2013. In April 2022, Kesaev was sanctioned by the EU, which identified that his tobacco holdings (among other business interests, including weapons production) were the source of significant revenue for the Russian government, contributing to the destabilisation of Ukraine. Reuters also suggested that these ties could make it difficult for PMI to extract itself from Russia, and indeed, earlier this year, PMI CEO Jacek Olczak admitted that Philip Morris may never sell its Russian business, citing the Kremlin’s terms on foreign companies’ asset sales and his duty to shareholders.

This is a stark contrast to earlier proposals for scaling back in Russia, particularly at a time when companies such as PMI and JTI continue to declare their commitment to becoming ethical companies.

### England: will the country ban disposable vapes?

Australia recently announced a full ban of all non-prescription vapes, which included a ban on single use disposable products. France is also considering a ban on disposables, and now the UK is reportedly considering following suit. Scotland has already announced plans to hold a public consultation on a ban and England is currently reviewing the evidence on youth vaping.

In the UK, in 2023, during even a short walk down the street, it is extremely rare not to come across at least one discarded vape: A colourful, easy to hold, plastic product, contaminated with e-liquid and containing a lithium-ion battery, discarded on the pavement. Some are intact and others are crushed or broken, leaving shards of plastic and the lithium-ion battery and plastic-coated metal wires concealed within. Left where they are, they will never biodegrade, but will eventually become microplastics and chemicals that will leak into our drains and pollute waterways and harm wildlife.

Manufacturers suggest that users contact their local recycling centres for recycling advice. Vaping products should not be thrown in the normal bin – in landfill, batteries leak, but they should not be placed in the plastic recycling bins either as they are comprised of more than plastic.

In the US, a 2020 survey found that 51% of young people were putting single-use vapes in the general waste bin, 17% in inappropriate recycling bins and 10% reported littering them.

A recent UK news report visited a recycling centre where it’s estimated only 30 % of disposable vapes end up. Even if all disposable vapes ended up at the right recycling point, an estimated 5 million disposable vapes are disposed of every week which would place a significant burden on recycling centres. While recycling is infinitely better than not recycling, focusing on recycling these products may be a welcome diversion tactic by an industry that is mass producing single use plastic products at time when we all need to reduce our environmental footprint.

In addition to the products’ environmental impact, the most recent figures from the UK Office of National Statistics suggested that 15.5% of 16–24 year-olds were using e-cigarettes in 2022, a significant increase from 11.1% in 2021.

Disposables are available in at point of sale in a variety of venues, including supermarkets, corner shops, and hardware stores. Although sales to under 18 s are prohibited, youths report few problems in acquiring them in stores. Youth-friendly flavours, and vibrant colours long since banned in tobacco products help entice young people.

The UK media is currently awash with stories on the dangers of disposable vapes for the environment and for contributing to a new epidemic of nicotine use among young people. England is famously pro-tobacco harm reduction and has had a historically permissive attitude to e-cigarettes. Will the UK regulate disposable products? Watch this space.

